# Performance of BVBlue Rapid Test in Detecting Bacterial Vaginosis among Women in Mysore, India

**DOI:** 10.1155/2014/908313

**Published:** 2014-01-12

**Authors:** Purnima Madhivanan, Karl Krupp, Tan Li, Kavitha Ravi, Julia Selezneva, Vijaya Srinivas, Anjali Arun, Jeffrey D. Klausner

**Affiliations:** ^1^Robert Stempel College of Public Health & Social Work, 11200 SW 8 Street, HLS 390W2, Miami, FL 33199, USA; ^2^Public Health Research Institute of India, 89/B, 2nd Cross, 2nd Main, Yadavairi, Mysore 560020, India; ^3^School of Public Health, 50 University Hall, No. 7360, University of California, Berkeley, CA 94720, USA; ^4^Division of Infectious Diseases, David Geffen School of Medicine, University of California, Los Angeles, CA 90095, USA

## Abstract

Bacterial vaginosis (BV) is the most common cause of abnormal vaginal discharge in reproductive age women. It is associated with increased susceptibility to HIV/STI and adverse birth outcomes. Diagnosis of BV in resource-poor settings like India is challenging. With little laboratory infrastructure there is a need for objective point-of-care diagnostic tests. Vaginal swabs were collected from women 18 years and older, with a vaginal pH > 4.5 attending a reproductive health clinic. BV was diagnosed with Amsel's criteria, Nugent scores, and the OSOM BVBlue test. Study personnel were blinded to test results. There were 347 participants enrolled between August 2009 and January 2010. BV prevalence was 45.1% (95% confidence interval (CI): 41.5%–52.8%) according to Nugent score. When compared with Nugent score, the sensitivity, specificity, positive predictive value, negative predictive value for Amsel's criteria and BVBlue were 61.9%, 88.3%, 81.5%, 73.7% and 38.1%, 92.7%, 82.1%, 63.9%, respectively. Combined with a “whiff” test, the performance of BVBlue increased sensitivity to 64.4% and negative predictive value to 73.8%. Despite the good specificity, poor sensitivity limits the usefulness of the BVBlue as a screening test in this population. There is a need to examine the usefulness of this test in other Indian populations.

## 1. Introduction

Finding effective methods to diagnose bacterial vaginosis (BV) has taken on increased urgency since BV was associated with a greater than 3-fold increased risk of female-to-male HIV-1 transmission and a doubling of risk for acquiring sexually transmitted infections [1–4]. BV has also been implicated in neonatal morbidity [5], preterm delivery [6–8], and low birth weight infants [9]. BV is usually diagnosed using Amsel's criteria [10] or Nugent scoring of Gram-stained vaginal smears [11] both of which require microscopy. In countries like India where access to laboratory services is often limited, BV is typically managed using a syndromic approach for vaginal discharge, a method with low sensitivity and specificity [12]. Point-of-care (POC) testing may improve diagnosis of BV by providing accurate results without laboratory or on-site microscopy [13].

Several different approaches have been used in POC testing for BV. Rapid assays detecting the presence of proline amino peptidase [14, 15] have been found to have high sensitivity and specificity. Others which rely on detection of trimethylamine and high vaginal pH have not been compared favorably with Amsel's criteria or Nugent scoring [16–18]. BVBlue, a chromogenic POC, diagnoses BV based on elevated levels of sialidase, an enzyme produced by anaerobic flora including *Bacteroides*, *Prevotella*, and *Gardnerella* species [19, 20]. In other studies, this approach has been shown to have excellent sensitivity, specificity, and predictive values in several populations [21–24].

This paper describes a study comparing the performance of BVBlue point-of-care test (OSOM BVBlue Test, Gryphus Diagnostics, Birmingham, AL, USA) with Amsel's criteria and Nugent scoring of Gram-stained vaginal smears among women attending a reproductive health clinic in Mysore, India.

## 2. Materials and Methods

### 2.1. Study Population

Between August 2009 and January 2010, a consecutive sample of 347 nonpregnant, sexually active women were recruited prospectively from a reproductive health clinic in Mysore, India, into a cross-sectional study comparing the performance of the BVBlue POC test to Amsel's criteria and Nugent scoring of Gram-stained vaginal smears. To be included in the study, participants were required to be 18 years of age or older, have had vaginal intercourse at least once in the previous three months, be willing to undergo a pelvic examination, and have a vaginal pH over 4.5. Vaginal pH was measured by placing a self-collected vaginal swab on a BDH pH test strip and comparing the color to a pH chart provided by the manufacturer (VWR International, West Chester, PA, USA). The pH strip was read by a research assistant responsible for assessing participant eligibility. The Committee for Protection of Human Subjects at University of California, Berkeley, CA, USA and the Institutional Review Board at Public Health Research Institute of India (PHRII), Mysore, India, approved the study protocol. All participants provided written informed consent to participate in the study.

### 2.2. Data Collection

After undergoing an informed consent process, trained interviewers collected information on demographics and reproductive/sexual health using a standardized questionnaire. Biological samples, questionnaires, and clinician checklists were labeled with a unique identifier to ensure confidentiality of participants.

### 2.3. Examination and Specimen Collection

A trained study clinician performed a pelvic examination and collected three swabs of vaginal fluid from the posterior fornix of the vagina in a random order to test for BV, *Trichomonas vaginalis*, and vaginal candidiasis. In addition, clinical signs from external and internal examination were recorded on a medical chart. Signs of vaginal discharge including amount, odor, color, and consistency were noted. Diagnosis was based on Amsel's criteria: presence of any three of four clinical features: a characteristic homogeneous white adherent vaginal discharge, a vaginal pH greater than 4.5, a positive amine test, and presence of 3–5 clue cells per high power field on wet-mount microscopy [10]. Symptomatic women diagnosed with BV by Amsel's criteria were treated according to standard Indian treatment guidelines.

### 2.4. Laboratory Assessment

All tests were performed by three trained laboratory research assistants in a blinded manner to prevent bias. Saline wet-mount preparation of vaginal fluid was examined microscopically within five minutes of collection for clue cells, motile trichomonads, and yeast buds/hyphae. The vaginal swab was placed in a test tube containing 3 drops of sterile normal saline at the time of pelvic examination by the clinician. After agitation, 1 drop of solution was placed on a glass slide, covered with a cover slip, and observed at 10x and 40x magnifications. Another drop of the saline solution was placed on a sterile glass slide with an added drop of KOH solution. The slide was used for detection of amine odor (whiff test). Following the whiff test, a cover slip was placed on the slide and read for detection of budding yeast or hyphae. Wet-mount examinations were part of the routine clinical care at this clinic and hence the time interval between specimen collection and microscopy was less than five minutes.

A second vaginal swab was placed in the BVBlue test vessel containing the chromogenic substrate of bacterial sialidase and the mixture was gently swirled. The BV test vessel containing the swab was left standing for 10 minutes. One drop of developer solution was added to the BV test vessel and the mixture was swirled gently again. Results were read immediately; a blue or green color in the BV test vessel or on the head of the swab was considered positive and a yellow color in the BV test vessel was considered negative. If the results were not blue/green or yellow, then the test was repeated. A positive result indicated an elevated level of sialidase activity and a negative result indicated a normal level.

The third vaginal swab was smeared on a glass slide and air-dried before fixing and Gram-staining at the PHRII laboratory. The swab was then used to inoculate InPouchTV culture kit (Biomed Diagnostic, White City, OR, USA), for detection of *T. vaginalis* infection. The swab was inserted in the upper chamber, agitated in the medium, and discarded, and the pouch was sealed. The contents of the upper chamber were immediately expressed into the lower chamber by rolling down. The pouch was then transported to the laboratory within four hours and placed in a 37°C incubator. It was read without opening the culture or sampling the contents for five days or until trichomonads were detected using a microscope at 10x and 40x magnification.

Each Gram-stained slide was scored by two different trained laboratory assistants masked to the other test results to minimize bias. In cases of discrepancy, the slide was then scored by a third reader blinded to the scores of the first two readers. The Nugent score is a standardized 0–10-point scoring system based on the presence of three bacterial morphotypes: large gram-positive rods (*Lactobacillus* spp.), small gram-negative or gram-variable coccobacilli (*Gardnerella* and anaerobic spp.), and curved gram-variable rods (*Mobiluncus* spp.) [11]. A Nugent score (NS) of 0–3 is classified as the presence of “normal” flora, 4–6 as the presence of “intermediate” flora, and 7–10 as BV. This method is still considered the gold standard for diagnosis of BV.

### 2.5. Quality Control

The expiration dates of all test kits were recorded before use. Kits were refrigerated between 2 and 8 degrees Celsius and kept out of direct sunlight. All BV vessels were stored inside the box as suggested by the manufacturer, and kits were brought to room temperature before use. For quality control purposes, test vessels were checked before use to ensure that they contained only a colorless liquid without sediments. The BVBlue test result was only reported if there was appearance of blue/green or yellow color in the testing vessel. We also conducted 10% random quality control checks by an experienced microscopist who has expertise in BV for Nugent's scoring and wet-mount preparation readings for clue cells and found high concordance.

### 2.6. Data Analysis

Data were entered and stored in Microsoft Access and analyzed using Stata 10.1 (Stata Corporation, College Station, TX). Proportions were compared using chi-square and Fisher's exact tests where appropriate, and 95% confidence intervals (CIs) were calculated. Participants were excluded from the analysis if complete clinical information or specimens were not available. Sensitivity, specificity, and predictive values were calculated using a traditional standard defined as Nugent score of 7–10 for BV positivity. Analyses were carried out in two ways: first, women with an NS of 4–6 (“intermediate” flora) were classified as negatives; second, women with “intermediate” flora were excluded, and the performance of the rapid test was calculated for each analysis. We also stratified the women based on their complaints into symptomatic (excess vaginal discharge, odor, burning, and itching) and asymptomatic cases to examine the performance of the rapid test. Sensitivity and specificity were calculated for each testing method using the following formulas:

Sensitivity: (number of true positives/(number of true positives + number of false negatives))∗100;

Specificity: (number of true negatives/(number of true negatives + number of false positives))∗100.

## 3. Results

### 3.1. Population Characteristics

**Figure 1:**
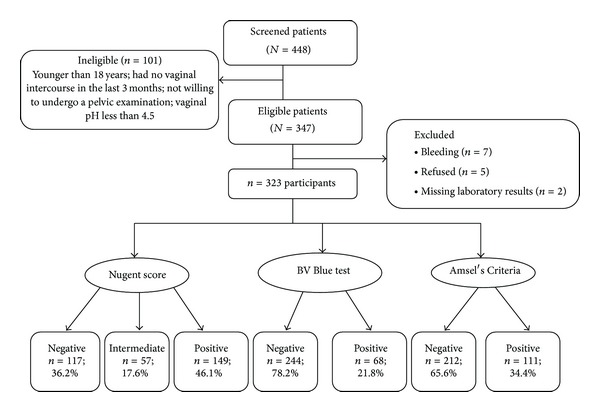
Enrolment figures for the study evaluating OSOM BVBlue Rapid Test among women in Mysore, India, between August 2009 and January 2010.

Figure 1 describes the number of women eligible who did not participate in the study and the reasons for not participating. Twelve participants were excluded because of missing laboratory results. (Seven were missing BVBlue results, and five Nugent score.) Among the total participants, 221 (71%) women reported symptoms of abnormal vaginal discharge, pruritus, burning, or odor at the time of enrollment. The characteristics of these participants are described in Table 1. The median age of the patients was 33 years and the vast majority (87%) reported their religion as Hindu. Eighty-seven (58.3%) women diagnosed with BV complained of vaginal symptoms, and 62 (41.6%) were asymptomatic.

### 3.2. Burden of BV among Participants

About 206 (63.7%) women had abnormal vaginal flora with an NS of 4–10, and 149 (46%) were diagnosed with BV (NS 7–10) (Figure 1). Using Amsel's criteria, 111 (35%) women were diagnosed with BV and 68 (21.9%) by the BVBlue POC test. *T. vaginalis *infection was common among women with BV (NS: 7–10) as compared to women without BV (18% versus 8.6%; *P* = 0.02).

### 3.3. Performance of BV Diagnostic Methods

A positive BVBlue test result was strongly associated with two of Amsel's criteria: the presence of clue cells (*P* < 0.0001) and amine odor (*P* < 0.0001). Table 2 shows the BVBlue POC test performance compared to wet-mount microscopy, Amsel's method, and Nugent scoring among a subset of women where women with intermediate flora were excluded from the analysis.  Table 3 shows the performance of BVBlue test as compared to Amsel's criteria and Nugent score. With BV positive being defined as NS of 7–10 and all other results considered negative including the intermediates (NS: 0–6), BVBlue performance was not very different among symptomatic women as compared to all women.

### 3.4. Test Sensitivity among Women with Symptoms

Among symptomatic women with complaint of any vaginal symptom, Amsel's criteria had the best sensitivity followed by wet mount microscopy and BVBlue with Nugent score being considered the gold standard. The BVBlue test performance did not improve (in terms of sensitivity and specificity) among symptomatic women as compared to its performance among all women (Tables 2 and 3).

### 3.5. Performance of BVBlue Combined with Amine Test

Used alone, a positive amine odor (whiff test) was highly specific (87.9%) but had a low sensitivity (59.7%). Combined use of a whiff test and the BVBlue POC test, however, improved the sensitivity of BV diagnosis to 64.4%. Diagnosing BV with a BVBlue POC test and/or amine test result was found to be the best performing approach with high sensitivity and minimal loss of specificity as compared to Amsel's criteria.

## 4. Discussion

Our study examined the performance of the BVBlue POC test as compared to Amsel's criteria and Nugent scoring of Gram stains among women with and without symptoms of BV. Our results showed that BVBlue test had poor sensitivity in detecting BV (38.1%) but was highly specific (92.7%) in a population of women attending a reproductive health clinic in Mysore, India. In previous research, BVBlue has been shown to perform well compared with conventional diagnostic methods for the diagnosis of BV in populations in Canada, Australia, Thailand, Malaysia, and China [21, 24–26]. Sensitivity ranged from 88% to 100% and specificity from 95% to 97.8% using Nugent Gram stain as a gold standard. The only study from North India showed that the BVBlue test had 97.6% sensitivity and 97.5% specificity as compared to Nugent score of Gram stain [23]. However, our findings were similar to another study that examined the performance of BVBlue for diagnosis of bacterial vaginosis in symptomatic and asymptomatic women in the US. That study found BVBlue test was less sensitive than Gram stain for diagnosis of BV and not statistically different from Amsel's criteria [27].

Because our results were substantially different from previous evaluations of the BVBlue, we considered and eliminated several alternative explanations for the difference. Our samples were collected by a trained study clinician who was an experienced obstetrician/gynecologist and processed within five minutes of the time that the physical examination was finished. So we believe that the quality of the specimens was adequate for evaluation. Our team had been extensively trained and recently completed several studies using Nugent score reading of several thousand Gram stains, so we ruled out systematic human error in the diagnosis of BV [28–33]. We had conducted quality control checks by an experienced microscopist for Nugent scoring and wet mount readings for clue cells and found high concordance. Finally, we ruled out manufacturing or handling problems with BVBlue kits by checking refrigerator logs and contacting the manufacturer to enquire about known quality issues with the manufacture or handling of the kits. Having examined and dismissed those alternative explanations, we speculate that the BVBlue POC kit was not sensitive in this population because of differences in the composition and diversity of the vaginal bacterial flora among women with BV in our sample. Recent studies using broad-range 16S rRNA gene PCR and pyrosequencing have shown that BV is a highly heterogeneous condition marked by greater species richness and diversity than previously thought, with no single species universally present [34]. Since the BVBlue kit operates on the principle that BV is associated with elevated levels of sialidases, it is possible that the vaginal biota of women with BV in our study population may have sialidase-negative *G. vaginalis* strains or contain a low number of anaerobic Gram-negative rods such as *Prevotella* spp. and *Bacteroides* spp. that are common sources of sialidases in BV. Previous studies have shown that sialidase activity was detected in only 75% to 84% of women [35, 36] with BV, suggesting that the presence of sialidase is not uniform. Studies have also demonstrated that the composition of BV flora varies by race/ethnicity, raising the possibility that BV-related bacteria in this population may be different from those found in other racial and ethnic groups [34, 37].

There are several limitations to this study. First, we did not evaluate the performance of the rapid test using molecular methods such as polymerase chain reaction (PCR) for diagnosis of BV. Currently, Nugent scoring of Gram stains continues to be the gold standard for diagnosis of BV in research studies, but it is possible that results might have been different if molecular diagnostic methods had been used. Second, corroborating low sialidase levels in the vaginal fluid of participants would have been useful in helping to explain the poor sensitivity of the BVBlue POC test, but it was beyond the scope of this study. Finally, we were not able to definitively establish the fitness of BVBlue kits used in the study since we were not provided with an external control by the manufacturer.

There is an important public health need for development of POC tests that facilitate diagnosis of BV in settings without adequate laboratory infrastructure. However, BVBlue test does not appear to be a good screening test in our population. Unfortunately, the effectiveness of different methods may depend on the bacterial composition of BV flora in different populations giving added importance to the need for wide evaluation of POC tests. Furthermore, given the heterogeneity of BV, culture-based methods for characterizing the vaginal biota may have to give way to more exacting molecular methods to detect and characterize a greater range of organisms. Additional research should also be conducted to analyze differences in vaginal biota of different populations in India and other parts of the world.

## Figures and Tables

**Table 1:** Characteristics of study population according to BV status diagnosed by Nugent score (7–10) among sexually active women in Mysore, India, between August 2009 and January 2010 (*N* = 323).

Characteristic	Total		BV Present^a^		*P* value
	*N*	(%)	*n*	(%)	
Total	323	100	149	46.1	
Age categories					0.13
18–25 yrs	34	10.5	15	44.1	
26–35 yrs	169	52.3	70	41.4	
≥36 yrs	120	37.2	64	53.3	
Education level					0.94
No education	133	41.2	64	48.1	
Up to 5 years of school	46	14.2	21	45.6	
6 to 9 years of school	75	23.2	33	44.0	
≥10 years	69	21.4	31	44.9	
Religion					0.14
Hindu	281	87.0	133	47.7	
Other	42	13.0	15	35.7	
Yeast infection on microscopy^b^					0.45
No	171	53.1	76	44.2	
Yes	151	46.9	73	48.3	
*Trichomonas vaginalis* by culture and/microscopy^b^					0.04
No	276	86.0	122	44.2	
Yes	45	14.0	27	60.0	

Note: Data are % (no.) of participants unless otherwise indicated.

^a^BV positive defined according to Nugent score of 7–10; ^b^denominator may vary because of missing data.

**Table 2:** Characteristics of the diagnostic tests used for detection of bacterial vaginosis among sexually active women with intermediate flora (Nugent score 4–6) excluded^a^.

Test	Bacterial vaginosis (Nugent score 7–10)							
	All women (*N* = 266)^b^				Symptomatic women (*N* = 156)^b^			
	Sens%	95% CI	Spec%	95% CI	Sens%	95% CI	Spec%	95% CI
Amsel's criteria	61	(55–67)	92	(88–95)	64	(57–72)	96	(93–99)
Wet-mount microscopy	46	(40–52)	98	(96–99)	49	(42–57)	97	(95–100)
BVBlue	38	(32–44)	95	(92–97)	37	(30–45)	95	(92–99)

^a^Nugent score of 0–3 considered negative, and 7–10 considered positive. Women with NS of 4–6 were excluded from this analysis. ^b^Number varies because of missing data. Sens: sensitivity; Spec: specificity; PPV: positive predictive value; NPV: negative predictive value. Amsel's criteria defined as any three of the four characteristics: vaginal pH > 4.5, presence of amine odor on addition of 10% potassium hydroxide (whiff test), presence of 3–5 clue cells per high power field on wet-mount microscopy, and homogenous vaginal discharge.

**Table 3:** Performance of BVBlue Rapid Test compared to the results of Amsel's criteria and Nugent score among sexually active women in Mysore, India, between August 2009 and January 2010.

	Variables	*N*	Sens% (95% CI)	Spec% (95% CI)	PPV% (95% CI)	NPV% (95% CI)
All women	Amsel's criteria^a^	323	51 (46–57)	94 (92–97)	82 (78–87)	78 (74–83)
	Nugent score^b^	266	38 (32–44)	95 (92–97)	90 (87–94)	54 (48–60)
Symptomatic women	Amsel's criteria^a^	188	52 (44–59)	95 (92–98)	85 (80–90)	79 (73–85)
	Nugent score^b^	156	37 (30–45)	95 (92–99)	91 (87–96)	54 (46–61)

^a^All women were included in this analysis comparing Amsel's criteria to BVBlue Rapid Test. Women with intermediate flora included as negatives (0–6) and NS of 7–10 considered BV positive. ^b ^Women with intermediate flora (NS of 4 to 6) excluded from this analysis comparing Nugent score to BVBlue Rapid Test. Sens: sensitivity; Spec: specificity; PPV: positive predictive value; NPV: negative predictive value.
